# Multiscale measurement of brain tissue and cell biomechanics using a mouse model

**DOI:** 10.52601/bpr.2025.240049

**Published:** 2025-12-31

**Authors:** Runke Wang, Suo Qian, Huijing Jin, Fuhua Yan, Guang-Zhong Yang, Yuan Feng

**Affiliations:** 1 School of Biomedical Engineering, Shanghai Jiao Tong University, Shanghai 200030, China; 2 Institute of Medical Robotics, Shanghai Jiao Tong University, Shanghai 200240, China; 3 National Engineering Research Center of Advanced Magnetic Resonance Technologies for Diagnosis and Therapy (NERC-AMRT), Shanghai Jiao Tong University, Shanghai 200240, China; 4 Department of Radiology, Ruijin Hospital affiliated to Shanghai Jiao Tong University School of Medicine, Shanghai 200025, China

**Keywords:** Brain biomechanics, Multiscale, Mouse model, Neuron, Astrocyte

## Abstract

The intricate structure of the brain at various scales is linked to its functional and biomechanical properties. Since understanding the biomechanical properties is vital for comprehending brain diseases, development, and injuries, measurements at different scales are necessary. Here we introduce methods to measure the biomechanical properties at both tissue and cellular levels using a mouse model. A specially designed magnetic resonance elastography system is introduced for imaging the mouse brain, enabling *in vivo* mapping of its shear modulus. Additionally, protocols for isolating and culturing primary neurons and astrocytes from the hippocampus and cerebral cortex of the mouse brain are presented. The nanoindentation technique using atomic force microscopy is employed to measure the biomechanical properties of individual cells. The results indicate that the storage/loss modulus of the mouse cerebral cortex and hippocampus are 8.07 ± 1.28 kPa / 3.20 ± 0.66 kPa and 6.60 ± 0.52 kPa / 2.52 ± 0.33 kPa, respectively. Meanwhile, the Young’s modulus for neurons and astrocytes is 470.88 ± 17.67 Pa and 681.13 ± 14.15 Pa, respectively. These findings demonstrate that the brain exhibits distinct biomechanical properties at different scales. The proposed methods offer general techniques for investigating the multiscale biomechanical properties of the brain.

## INTRODUCTION

As one of the most complex organs, the brain has a distinct anatomical hierarchy, with inherent multiscale structures. At the tissue level, different brain regions have specific functionalities connected with each other. Brain tractography using diffusion tensor imaging (DTI) can trace the axonal fibers, showing the anatomical connections of different brain regions. The activation level indicated by the oxygen consumption constructed using functional magnetic resonance imaging (fMRI) maps out the functional connectivity of the brain. At the cellular level, neurons and glia cells form a complex network, presenting the cellular structure for the anatomical and functional connectivity at the tissue level. The multiscale feature of the brain has inspired multiscale modeling and measurement of the brain from both the anatomical (Park *et al.* 2024), biochemical (Xie *et al.* 2024), and functional perspectives (D’Angelo and Jirsa 2022).

It is well known that biomechanical properties are crucial to understand the disease, development, and injury of the brain (Goriely *et al.* 2015). Understanding the biomechanics of the brain at multiscale levels helps in modeling and computation of the brain (Prabhu and Horstemeyer 2022), as well as gaining insights into the mechanobiology of the brain disease (Procès *et al.* 2022; Tyler 2012). For example, with the biomechanics of the axonal fibers, the upscale modeling of white matter can provide a more accurate and detailed simulation of its behavior (Saeidi *et al.* 2023). Studies have also shown that, at the tissue level, neurodegeneration is closely related to the decrease of tissue stiffness (Feng *et al.* 2024). At the same time, the changes of biomechanical properties of neurons are also observed with the degenerative specific proteins (Li *et al.* 2023). However, how the neuronal cell mechanics are connected with the tissue level changes is still largely unknown. One of the key issues to address this challenge is a consistent and continuous method to measure the biomechanical properties at both the tissue and cellular levels.

For tissue-level measurement, classical *ex vivo* testing has been used widely, such as compression and tension tests (Eskandari *et al.* 2020; Rashid *et al.* 2012), simple/dynamic shear tests (Destrade *et al.* 2015; Rashid *et al.* 2013), and indentation tests (Budday *et al.* 2015; Feng *et al.* 2017; MacManus *et al.* 2017; Qiu *et al.* 2020; van Dommelen *et al.* 2010). However, *in vivo* measurements are also crucial for multiscale brain analysis, as they provide viscoelasticity data for longitudinal studies and enable the isolation of neuronal cells for further cellular-level measurements. Magnetic resonance elastography (MRE) offers an ideal approach for the biomechanical characterization of brain tissue *in vivo* (Sack 2022). Originally used for liver fibrosis diagnosis, MRE has been used for measuring the biomechanical properties of the brain for both humans (Pavuluri *et al.* 2023; Streitberger *et al.* 2019; Svensson *et al.* 2021) and animals (Feng *et al.* 2016; Palotai *et al.* 2022). With noninvasive and no injection needed, MRE can keep the original physiological state of the brain before the next step of cellular measurement. However, specific actuators and setups are needed for imaging of the mouse brain.

For measurement of biomechanical properties of cells, typical techniques include nanoindentation using atomic force microscopy (AFM) (Hu *et al.* 2020; Pei *et al.* 2019), microfluidic methods such as shear flow deformability cytometry and extensional flow deformability cytometry (Urbanska *et al.* 2020), micropipette aspiration (Ding *et al.* 2018), magnetic and optical tweezer (Guck *et al.* 2001; Hao *et al.* 2020). AFM offers a viable and efficient means of characterizing neuronal cells, such as neurons with extended axons. By selecting specific indentation tips, various parts of the neuronal cells can be measured. Furthermore, compared to other measurement techniques, the preparation of nanoindentation for neuron cells is relatively simpler, allowing for direct stiffness measurement of a single cell without any special sample treatment. Additionally, AFM boasts the ability to measure in air, liquid, vacuum, and other environments, enabling real-time dynamic observation of living cells in a liquid setting and allowing for the characterization of local biophysical properties at the nanoscale.

In this methodological study, we proposed multiscale measurement techniques at both tissue and cellular levels for the brain using a mouse model. We introduced a custom-built MRE device for *in vivo* assessment of the biomechanical properties of brain tissue. Additionally, we outlined methods for isolating and preparing neuronal cells for nanoindentation. The proposed multiscale characterization method is not limited to the brain and neuronal cells; it can also be applied to other suitable tissues and cells in mechanobiology research.

## RESULTS

### Biomechanical mapping of mouse brain by MRE

**Figure 1:**
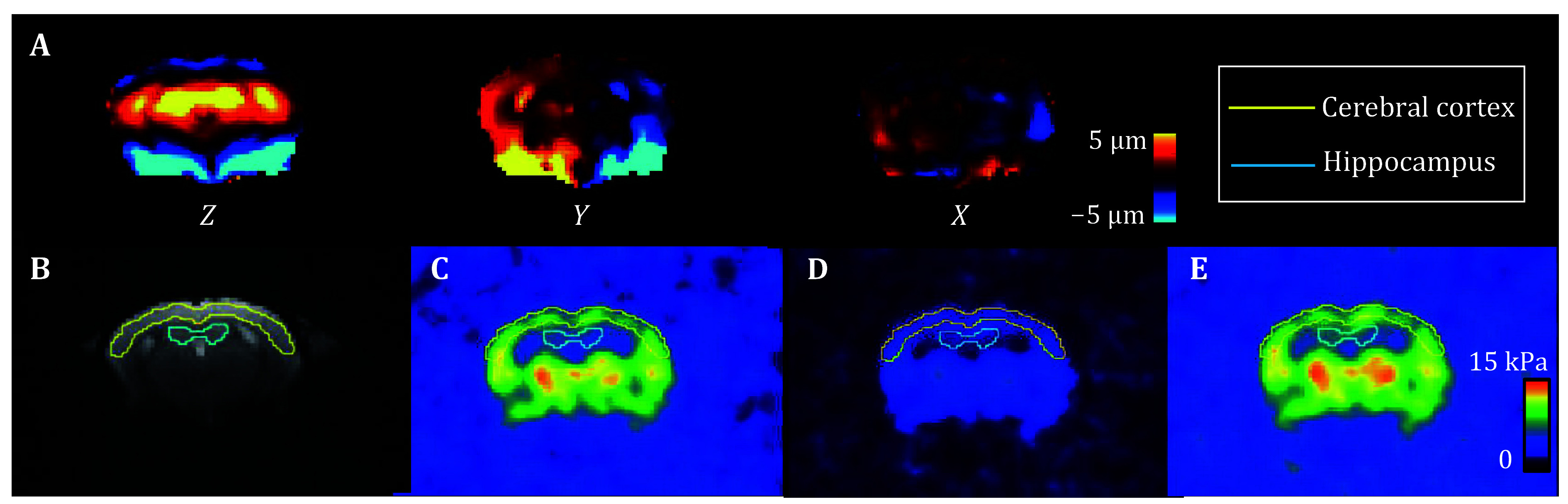
**A** Shear wave propagation in a mouse brain was recorded along three orthogonal axes using MRE at a frequency of 1.0 kHz, capturing wave images. **B** The cerebral cortex and hippocampus regions were delineated using the magnitude images acquired by the MRE sequence. Panels C, D and E are maps of the storage modulus (G’) (**C**), loss modulus (G’’) (**D**), and shear stiffness (|G*|) (**E**), respectively

Clear shear wave propagation was observed in the mouse brain (Fig. 1A). The magnitude of the wave was ~5 μm. The region of interest (ROI) of the cerebral cortex and hippocampus were delineated manually on the magnitude image for analysis of their biomechanical properties (Fig. 1B). For the wild type mouse, the storage/loss modulus of the cerebral cortex was 8.07 ± 1.28 kPa / 3.20 ± 0.66 kPa, while the hippocampus was 6.60 ± 0.52 kPa / 2.52 ± 0.33 kPa. The shear stiffness of the cerebral cortex and the hippocampus were 8.68 ± 1.42 kPa and 7.06 ± 0.59 kPa, respectively.

### Measurement of cell stiffness by AFM

**Figure 2:**
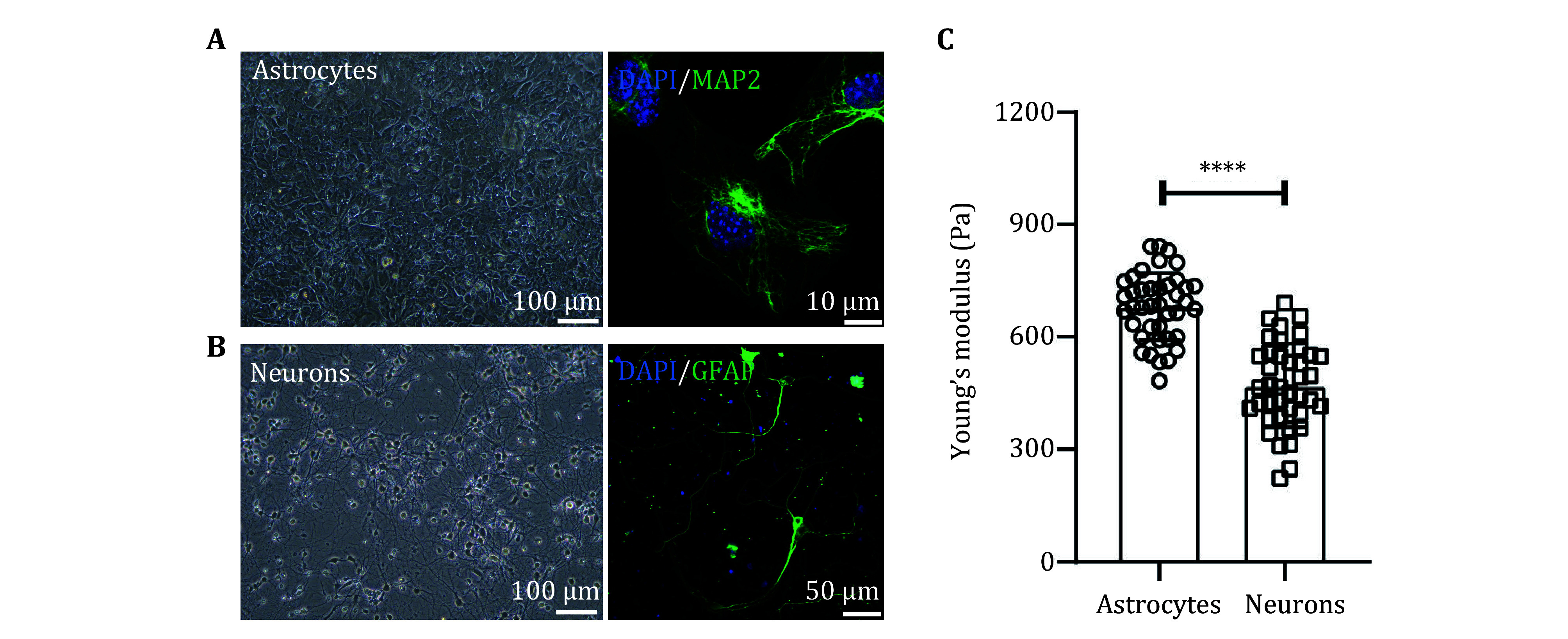
The stiffness of primary astrocytes and neurons was measured using AFM. Representative bright-field images of astrocytes (**A**) and neurons (**B**) *in vitro* are shown alongside an immunofluorescent image staining the nuclear marker DAPI (blue) and the astrocyte/neuron marker GFAP/MAP2 (green). The scale bar for the bright-field images is 100 µm, and the scale bar for the immunofluorescent images is 50 µm. **C** A comparison of Young’s modulus, measured using AFM, between astrocytes and neurons revealed a significant difference (*N* = 40 cells/group, ****p < 0.0001)

The primary astrocytes were isolated *in vitro* and their identity was confirmed using GFAP (Fig. 2A), while neuron cells were identified with MAP2 (Fig. 2B). The stiffness of astrocytes and neurons was measured using AFM. The results indicated that astrocytes exhibited a stiffness of approximately 681.13 ± 14.15 Pa, whereas neurons demonstrated a stiffness of about 470.88 ± 17.67 Pa. Consequently, astrocytes were significantly stiffer than neurons (Fig. 2C).

## DISCUSSION

In this study, methods for assessing the biomechanical properties of the brain at both tissue and cellular levels were proposed. Specifically, a small animal MRE system was developed to achieve *in vivo* biomechanical mapping of brain tissue at the tissue level. Additionally, the same mouse brain could be dissected for biomechanical measurements at the cellular level. Furthermore, methods for preparing and measuring both neurons and astrocytes were introduced.

To generate shear waves in the mouse brain, various MRE systems have been suggested, utilizing either piezoelectric (Feng *et al.* 2013) or electromagnetic (Chatelin *et al.* 2015; Schregel *et al.* 2017) actuators. In the case of piezoelectric MRE systems, the setup is akin to the proposed system, where a piezoelectric actuator is directly linked to the mouse's incisors via a bite bar (Feng *et al.* 2013). However, positioning the actuator proximal to the magnet bore for effective actuation can lead to magnetic field inhomogeneity and potential bulk motion. To mitigate these issues, the proposed system uses remote actuation, ensuring minimal vibration transmission loss. Unlike the electromechanical-acoustic actuator, which uses a connecting bar and piston through the RF coil to transmit vibrations to the mouse's head in a supine position (Chatelin *et al.* 2015), the proposed setup is simpler to install and does not restrict coil selection. This aligns with the nose cone setup for vibration transmission (Schregel *et al.* 2017), which offers ease of installation and adaptability.

Using the proposed MRE system, the estimated shear stiffness of the cortex for the WT mouse was slightly higher (8.7 kPa) than the reported average (6.7 kPa), while the shear stiffness of the hippocampus (7.1 kPa) was comparable to the reported average (5.6 kPa) (Bigot *et al.* 2018). This difference can be attributed to the age disparity of the mice used in the studies. Studies have demonstrated that cortical stiffness rises with age (Munder *et al.* 2017). The 15-month-old mouse used in this study was older than those used in previous research (Bigot *et al.* 2018), potentially leading to a higher stiffness measurement. Additionally, the inversion algorithm employed may also affect the results (Bertalan *et al.* 2019). For instance, the stiffness of the cortex estimated using the Algebraic Helmholtz Inversion (AHI) algorithm exhibited a ~24% decrease compared to that estimated by the tomoelastography (k-MDEV) method (Bertalan *et al.* 2019). In a prior comparative study, we also observed a ~6%/~2% variation in stiffness estimation when using the Traveling Wave Expansion-based Neural Network (TWENN) algorithm (Ma *et al.* 2023) compared to AHI/k-MEDV.

Mouse model provides an indispensable tool to investigate the brain, while MRE provides a non-invasive imaging method to study the biomechanics of the brain and its related diseases. For brain tumors, studies have shown that tumor types such as meningioma and pituitary tumors can affect the biomechanical properties of the brain (Cohen-Cohen *et al.* 2021; Murphy *et al.* 2013). Using mouse brain MRE, it was found that tumor mechanics changed after radiotherapy (Feng *et al.* 2016) and immunotherapy-monitoring can be achieved with response-related tumor mechanics (Streibel *et al.* 2024). For neurodegenerative diseases such as Alzheimer’s Disease (AD) and Parkinson’s Disease (PD) (Jin *et al.* 2024; Yang *et al.* 2022), a significant decrease of tissue stiffness was observed using MRE (Lipp *et al.* 2018; Murphy *et al.* 2016, 2019; Streitberger *et al.* 2012). Furthermore, many mouse models have been developed to study neurodegenerative diseases (Long *et al.* 2022). Using a mouse model, multiscale measurement of biomechanical and biochemical analysis could be carried out using high-resolution microscopy and immunohistochemistry (Hain *et al.* 2016; Palotai *et al.* 2022; Schregel *et al.* 2012). Thus, mouse-model MRE offers a unique and powerful tool to study the brain and its diseases.

Measuring the biomechanical properties at the cellular level is essential for understanding tissue-level behavior and changes. Cell stiffness significantly influences cell fate and function and is closely associated with disease development (Chiang *et al.* 2013). As an effective tool for measuring the modulus of individual cells, AFM is widely utilized in brain disease research. AFM can be employed for liquid biopsy to assess glioblastoma malignancy in mice (Thakur *et al.* 2021). In clinical settings, AFM can evaluate thrombus characteristics of acute ischemic stroke in combination with infrared spectroscopy and Raman spectroscopy (Blat *et al.* 2019). AFM also effectively characterizes the mechanical properties of the extracellular matrix (Hartmann *et al.* 2024) and cells during disease progression, playing a pivotal role in advancing neurobiological mechanics research.

At the cellular level, we selected neurons and astrocytes as representative neuronal cells to measure their biomechanical properties. Neurons serve as the foundation for brain information transmission and functional operations, and their biomechanical properties are crucial for brain growth and development. The external mechanical environment influences the aggregation (Spedden and Staii 2013), differentiation (Kayal *et al.* 2019), and maturation (Mattiassi *et al.* 2023) of neurons. Furthermore, alterations in the biomechanical properties of neurons can serve as indicators of different tissue development stages (Iwashita *et al.* 2014). Studies have demonstrated that the Young's modulus of neurons, measured using AFM, typically ranges between 150–650 Pa (Chen *et al.* 2016; Iwashita *et al.* 2014; Spedden and Staii 2013), aligning with our findings. The variation in neuron mechanics measurements can be attributed to various factors, including testing methodologies, as well as the origin and state of the neurons (Lu *et al.* 2006).

Astrocytes are the most abundant cell type in our brain (Freeman 2010), and their stiffness and functional response are influenced by mechanical forces (Gomez-Cruz *et al.* 2024). Studies have indicated that the stiffness of astrocytes impacts neurogenesis and cognitive brain function (Chi *et al.* 2022). The Young's modulus of primary astrocytes, measured using AFM, has been reported to range between approximately 320 Pa (Atashgar *et al.* 2022), 600 Pa (Chen *et al.* 2016) and 714 Pa (Gomez-Cruz *et al.* 2024), aligning with our findings. However, some studies have reported higher moduli (Atashgar *et al.* 2022), such as 2.2 kPa (Efremov *et al.* 2011), 3.8 kPa (Lee *et al.* 2015), and even 14 kPa (Ghézali *et al.* 2024). This discrepancy may be attributed to several factors. Firstly, the stiffness of the nucleus is about 2–10 times greater than the cell body (Zwerger *et al.* 2011). Nanoindentation with a large indentation depth may compress the nucleus extensively, and contribute to the overall cell stiffness. Secondly, culturing conditions can affect the measurement, as the Young's modulus of cells increases with increasing substrate stiffness (Atashgar *et al.* 2022). Thirdly, astrocyte stiffness varies with their developmental stage, with more mature astrocytes exhibiting greater stiffness (Lee *et al.* 2015). Finally, factors such as the probe size used in AFM measurements, the indentation frequency of the needle, and the data processing model can all influence the estimated cell stiffness (Müller *et al.* 2020).

The complexity of brain structures and physics is demonstrated by the variations in biomechanical properties at both tissue and cellular levels. Apart from neuronal cells, the extracellular matrix plays a crucial role in shaping the biomechanical properties of brain tissue, potentially contributing to the tissue-level stiffness that is one magnitude higher. Furthermore, *in vivo* measurements at the tissue level using MRE are frequency-dependent. As tissue stiffness rises with increasing frequency (Okamoto *et al.* 2011; Qiu *et al.* 2021), measurements at 1 kHz could yield relatively higher absolute values compared to quasistatic methods like indentation. However, when neurons and astrocytes were isolated from the hippocampus and cortex regions respectively, consistent biomechanical differences were observed. Specifically, astrocytes were found to be approximately 42% stiffer than neurons, and the cortex was approximately 45% stiffer than the hippocampus. Future studies will explore whether neuronal cells play a significant role in influencing these biomechanical properties.

## CONCLUSION

We introduced methods to measure the biomechanical properties of the brain at both tissue and cellular levels using a mouse model. At the tissue level, we proposed an MRE system to measure the stiffness of the mouse brain *in vivo*. Piezoelectric actuators transmitted vibrations to the mouse brain with custom-built animal support systems. Additionally, we introduced procedures for isolating and culturing neurons and astrocytes, along with protocols for nanoindentation of cells using AFM. The results showed that the shear modulus at the tissue level was approximately ten times higher than that at the cellular level, demonstrating a clear frequency-dependent behavior of the brain and its complex biomechanical structures. The methods introduced could serve as valuable tools for investigating the biomechanics of the brain at different scales.

## MATERIALS AND METHODS

### MRE actuator for small animal

**Figure 3:**
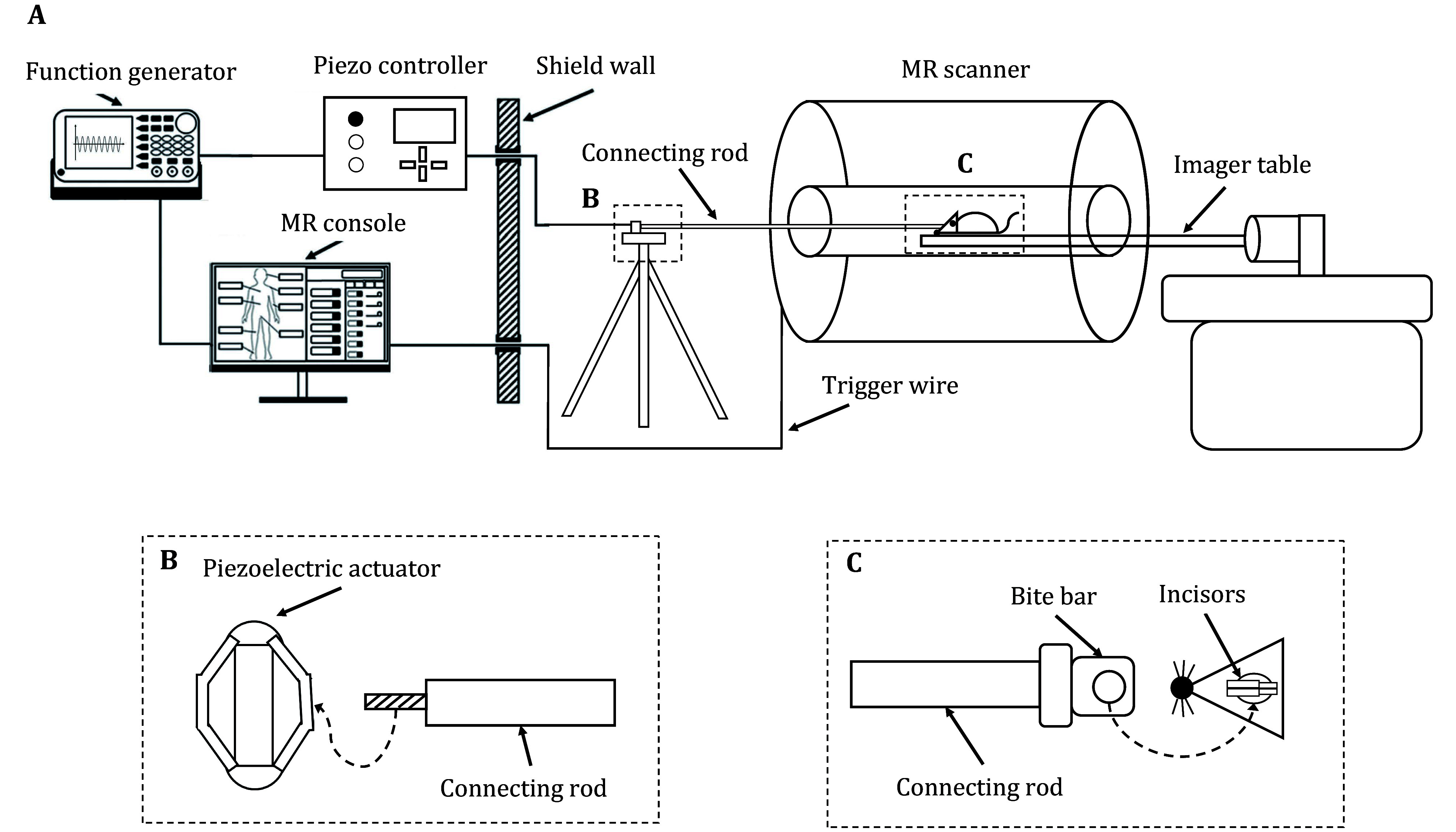
**A** An overview of the MRE actuator for small animals. The MR console starts an MRE scan and sends a trigger signal to the function generator to generate a sinusoidal signal. The piezoelectric actuator then generates a sinusoidal vibration and transmits it to the mouse brain through a connecting rod. **B** A rigid connection between the piezoelectric actuator and the connecting rod is established using a screw. **C** A rigid connection between the mouse’s incisors and the connecting rod is established using a bite bar

A custom-designed MRE device for small animals is built to generate sinusoidal waves with specific frequencies to the mouse brain (Fig. 3). A function generator (DG1000Z, RIGOL Technologies, Suzhou, China) receives the trigger signal from the MR scanner and generates sinusoidal alternating current (AC) with desired frequencies. A modular piezo controller (E01.C1, CoreMorror, Harbin, China) amplifies the AC and drives the piezoelectric actuator (40A5, CoreMorror, Harbin, China) to produce vibrations. A rigid connecting rod (~2 m) made of Poly-Ether-Ether-Ketone (PEEK) transmits the waves to the brain without affecting the homogeneity of the magnetic field. At one end of the rod, a bite bar is hooked over the mice’s incisor. At the other end, the piezoelectric actuator is firmly attached with a screw. A non-magnetic tripod is used to hold the piezoelectric actuator, making sure the connecting rod and the imager table are all aligned at a uniform height. This setup is able to produce vibration at a specified frequency to the mouse brain and synchronized with the MRE sequence.

### Measurement protocol for animal MRE

One 15-month-old (mo) female wild type C57BL/6J mice were used for the MRE test. The animal experimental procedures were approved by the Institutional Animal Care and Use Committee (IACUC) of Shanghai Jiao Tong University, and were conducted in accordance with the US National Research Council’s Guide for the Care and Use of Laboratory Animals. The animal studies were reported following the ARRIVE guidelines.

MRE scans were performed on a 9.4 T small animal MR scanner with a three-channel head coil (uMR 9.4T, United Imaging Healthcare, Shanghai, China). The mice were induced to anesthesia using 3% isoflurane in 100% O_2_ and transferred to the animal holder of the MR scanner. The bite bar was attached to the mice’s incisors. The connecting rod was rigidly connected to the piezoelectric actuator (Fig. 3). Anesthesia of the mouse was achieved by sending 1%–1.5% isoflurane in 100% O_2_ through the nose cone to the mouse.

At the start of the scan, a trigger signal was dispatched from the MR scanner to the custom-built MRE actuator, prompting it to generate waves that were transmitted to the mouse brain. A Gradient Recalled Echo (GRE)-MRE sequence was employed to capture wave images utilizing a Motion Encoding Gradient (MEG). The MEG encoded the wave information into MRI phase images, as outlined in Eq. 1. The correlation between the shear stiffness and the steady-state wave was expressed in Eq. 2. The imaging parameters were set as: TR/TE = 200/15 ms; FOV = 19.2 × 19.2 mm^2^; matrix = 96 × 96; resolution = 0.2 × 0.2 mm^2^; slice thickness = 0.3 mm. Waves at a frequency of 1.0 kHz were generated and acquired for the mouse. Four phase offsets of the wave were sampled, while six motion directions (±*x*, ±*y*, ±*z*) were encoded. The acquired wave images were unwrapped for each slice using the dual-DC optimization algorithm (Ma *et al.* 2023). The shear stiffness maps were estimated from the unwrapped wave images using a TWENN algorithm (Ma *et al.* 2023).

1 $ \phi=\int_{t_0}^{t_0+T}{\boldsymbol{G}}\left(t\right)\cdot{\boldsymbol{u}}\left(t\right){\sin}\left(\omega t+\psi\right), $

2 $ \rho \ddot{\boldsymbol{u}}=\left(\lambda +\mu \right)\nabla \left(\nabla \cdot  \boldsymbol{u}\right)+\mu \mathrm{\Delta }\boldsymbol{u} $

where *ϕ* represents the phase, ***G***(*t*) is the amplitude of the MEG, *t*_0_ and *T* denote the start time and duration of the MEG, respectively; ***u***(*t*) is the amplitude of the external wave, *ω* and *ψ* are the frequency and initial phase of the external wave, respectively; *ρ* is the density of the tissue. The notation $ \ddot{(\cdot )} $ denotes the second order temporal differentiation, while $ \nabla $ represents the Laplacian operator. *λ* is the Lamé constant and *μ* is the shear modulus. In the case of incompressible tissue, *λ* can be disregarded.

### Primary neurons and astrocytes culture

Primary neurons and astrocytes were isolated from the hippocampus and cerebral cortex of newborn C57BL/6J mice, respectively. The newborn mice were sterilized with 75% alcohol and immediately executed. Their skulls were peeled off on the ice to take out the brain tissue. Gently removed the cerebellum, nucleus and meninges, dissected the cerebral cortex and hippocampus from the brain, and washed them three times with Dulbecco's modified eagle medium (DMEM, Meilunbio, Shanghai, China) precooled at 4°C. The cerebral cortex and hippocampus were trypsinized with 3 mL of 0.25% (*v*/*v*) trypsin (Meilunbio, Shanghai, China) at 37°C for7 min and 5 min, respectively, suspended with 3 mL complete medium (DMEM + 10% (*v*/*v*) FBS (Gibco, Carlsbad, NM, USA) + 1% (*v*/*v*) penicillin streptomycin antibiotics (HyClone, Logan, UT, USA). The cerebral cortex was filtered with a 70-μm filter (Millipore, Burlington, MA, USA) to obtain astrocytes, and the hippocampus was filtered with a 40-μm filter (Millipore, Burlington, MA, USA) to obtain neurons. Those dissociated cells were plated on 6-well plates coated with poly-D-lysine (PDL, Sigma Aldrich, USA) and cell slides at a density of 6 × 10^5^ cells/well, and cultured with complete culture medium in a humidified incubator at 37°C with 5% CO_2_. The mixed glial cells culture medium was replaced with a complete medium every three days. Astrocytes were obtained on the 10^th^ day after shaking off microglia. Neurons were treated with complete culture medium for 2 h before changing to neuron culture medium (Neurobasal (Gibco, Carlsbad, NM) + 2% (*v*/*v*) B27 (Gibco, Carlsbad, NM, USA) + 1% (*v*/*v*) penicillin streptomycin antibiotics (HyClone, Logan, UT, USA)). Half of the neuron culture medium was changed every three days, and neurons were obtained on the 7^th^ day.

### Nanoindentation using AFM

**Figure 4:**
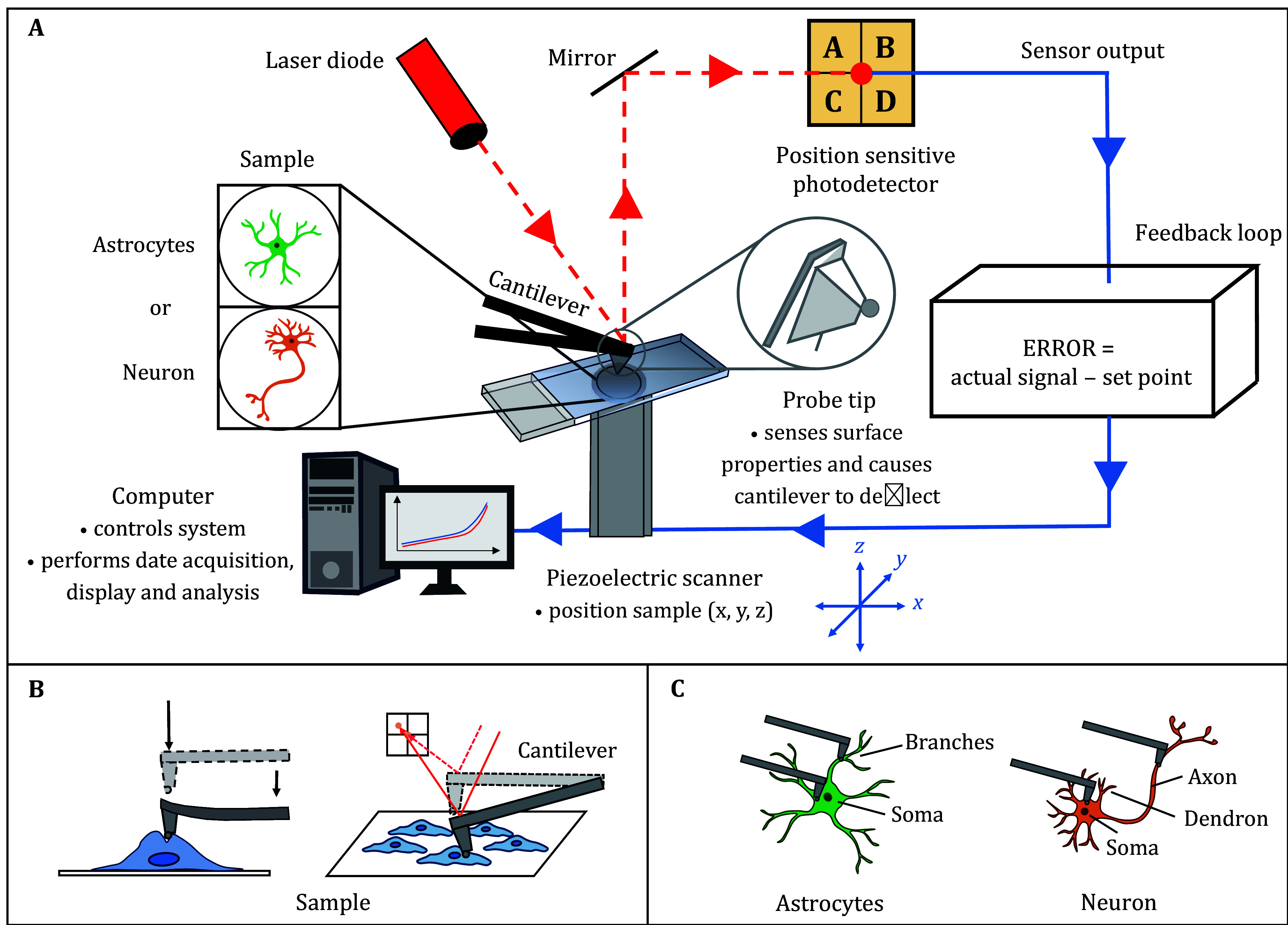
**A** Atomic force microscope general components and their function. **B** Schematic diagram of AFM detection of cell modulus. **C** Schematic diagram of measuring different parts of astrocytes and neurons by AFM

The mechanical properties of primary neurons and astrocytes were measured by nanoindentation using atomic force microscopy (FastScan Bio, Bruker, USA). During indentation, a laser beam is directed from the laser diode onto the cantilever of the probe and then reflected onto the photodetector to record the initial position. As the probe tip interacts with the sample surface, the cantilever deflects, causing the reflected laser beam to shift. This change in position is recorded as a new signal by the photodetector. The difference between the initial and new signals is fed back into the system to calculate the Young’s modulus, which serves as an indicator of cell stiffness (Fig. 4).

Young's modulus of cells was calculated based on Hertz model:

3 $ E=\frac{4}{3}\frac{1-{\nu}^{2}}{F}{\delta }^{-3/2}{R}^{-1/2}, $

where *F* is the indentation force, *δ* is the corresponding indentation displacement, and *R* is the radius of the spherical probe. Since cells can be considered incompressible, a Poisson's ratio of *ν* = 0.5 was used.

In the nanoindentation experiment of primary astrocytes and neurons, we recorded the force-displacement curve of cells through contact mode AFM with microsphere colloidal probes (MLCT-O10, *R* = 6 μm, *k* = 0.1 N/m, back side coating Ti/Au 45 nm, Bruker, USA). Cell slides covered with PBS were adsorbed on the sample table by vacuum adsorption. Move the microsphere colloidal probe to gently contact the liquid surface and focus on the position of the cell to be measured. Adjust the initial position of the laser to focus on the probe tip and set the probe insertion parameters to the ramp size of 1.5 μm, the ramp rate of 1.04 Hz and the deflection sensitivity of 33 nm/V. The recorded force-displacement curve was analyzed by NanoScope software (Bruker, Billerica, MA, UAS) to obtain the Young’s modulus.

## Conflict of interest

Runke Wang, Suo Qian, Huijing Jin, Fuhua Yan, Guang-Zhong Yang and Yuan Feng declare that they have no conflict of interest.
